# Functional Reproductive and Developmental Traits Differences Between Two *Macrocystis* Ecomorphs: Importance of Neutral Lipids

**DOI:** 10.1002/ece3.70899

**Published:** 2025-02-12

**Authors:** Camilo Rodríguez‐Villegas, Alejandro H. Buschmann, Mayra A. Barrios, Sandra Pereda, Carolina Camus, Pamela Fernández, María C. Hérnandez‐González, Ángela M. Baldrich, Cynthia Urrutia, Ailen M. Poza, Karina Villegas, Camila Martínez, Jaime Vargas

**Affiliations:** ^1^ Centro i~mar Universidad de Los Lagos Puerto Montt Chile; ^2^ CeBiB Universidad de Los Lagos Puerto Montt Chile; ^3^ Millenium Nucleus MASH Universidad de Los Lagos Puerto Montt Chile; ^4^ Facultad de Ciencias Marinas Universidad Nacional del Comahue Río Negro Argentina; ^5^ Departamento de Recursos Naturales y Medio Ambiente Universidad de Los Lagos Puerto Montt Chile; ^6^ Scientific and Technological Bioresource Nucleus (BIOREN) Universidad de La Frontera Temuco Chile

**Keywords:** *Macrocystis* ecomorphs, neutral lipids, reproductive and early developmental traits, sporophytes, zoospores

## Abstract

The kelp genus *Macrocystis* presents populations recognizable by distinct morphological traits, which has raised discussions on its taxonomical status. Recently, whole genome sequencing arose global evidence of *Macrocystis* ecomorphs “*integrifolia*” and “*pyrifera*” being genetically distinct. In the southern hemisphere, both ecomorphs maintain a separated distribution that coincides, without overlap, in the area of 33° S. Besides the fact that both ecomorphs are interfertile, at least under laboratory conditions, several differences in their reproductive strategies and early developmental traits have arisen in over 20 years of ecological and ecophysiological studies. In this study, we evaluated the content of neutral lipids in zoospores of both ecomorphs as a proxy of the required energy to swim and settle. The finding showed that zoospores of “*integrifolia*” ecomorph have a significantly lower level of neutral lipids than the southern “*pyrifera*” ecomorph. This correlates with the lower motility of the cells and lower germination capacity in the ‘integrifolia’ ecomorph, which has been consistently seen over the years. Further, we have seen a highly consistent pattern of a higher zoospore production, germination rate, sexual reproductive success, and early developmental performance (growth and survival) of juvenile sporophytes of “*pyrifera*” compared to “*integrifolia*.” Hence, both ecomorphs show, in addition to genetic and morphological differences, differential reproductive functional traits consistent in time and space that further support the hypothesis of an ongoing separation of these two *Macrocystis* ecomorphs.

## Graphical abstract

The kelp genus Macrocystis presents populations recognizable by distinct morphological and genetic traits, which has raised discussions on its taxonomical status. In addition, this study shows consistent functional and developmental traits (e.g., neutral lipid in zoospores and germination, sexual reproduction success, growth rate) between “pyrifera” and “integrifolia” ecomorphs in the costa of Chile. This evidence supports the hypothesis of an ongoing separation of these two Macrocystis ecomorphs.

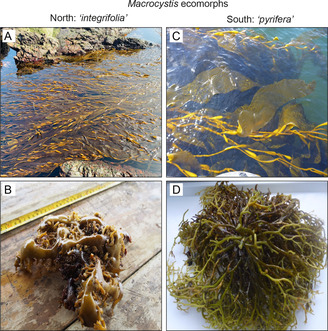

## 1 Introduction

The kelp *Macrocystis pyrifera* distributed along the East Pacific coastline, presents well‐separated populations recognizable by distinct morphological traits and genetic differentiation, raising discussions on its taxonomical status (Coyer, Smith, and Andersen 2001; Demes, Graham, and Suskiewicz 2009; Macaya and Zuccarello 2010; Camus, Faugeron, and Buschmann 2018; Gonzalez, Alberto, and Molano 2023). These populations were described as distinct species based on the holdfast and blade morphologies. However, due to low genetic divergence (Macaya and Zuccarello 2010) and high phenotypic plasticity, they were synonymized into one species (Demes, Graham, and Suskiewicz 2009). Nevertheless, whole genome sequencing arose evidence for *Macrocystis* ecomorphs “*integrifolia*” and “*pyrifera*” as being genetically divergent, raising the taxonomic question again (Gonzalez, Alberto, and Molano 2023). Camus, Faugeron, and Buschmann (2018) using microsatellites showed that genetic clusters found along the Chilean coast were correlated to its morphological diversity; hence, morphology and genetics may respond to the same environmental drivers. Moreover, comparative studies on both ecomorphs have shown differences in their reproductive strategies, such as zoospore activity, germination capacity, and gametophyte development rate (Buschmann et al. 2004; Camus et al. 2021).

As zoospore activity and germination depend on the presence of neutral lipids reserves (hereafter NL), we hypothesize that both ecomorphs, “*integrifolia*” and “*pyrifera*,” found respectively in the north (18° to 32° S) and south (32° to 56° S), have differentiated reproductive functional traits. In this sense, zoospores with higher NL may develop more viable gametophytes, as NL is essential for the swimming and germination of zoospores (Brzezinski, Reed, and Amsler 1993; Reed et al. 1999). By considering the relevance of NL on the viability of early stages of the haploid phase of Giant kelp, we measured the proportion of zoospores that show NL presence in the cells. After germination, the gametophytes rely on their photosynthetic performance, which is determined by environmental factors such as pH, temperature, and light (e.g., Labbé et al. 2024). By combining this information with 20 years of studying developmental and reproductive traits in these populations in the Southern East Pacific, we intend to add valuable information for future discussions on the evolution of the *Macrocystis* genus.

## 2 Materials and Methods

### 2.1 Field Sampling

**FIGURE 1:**
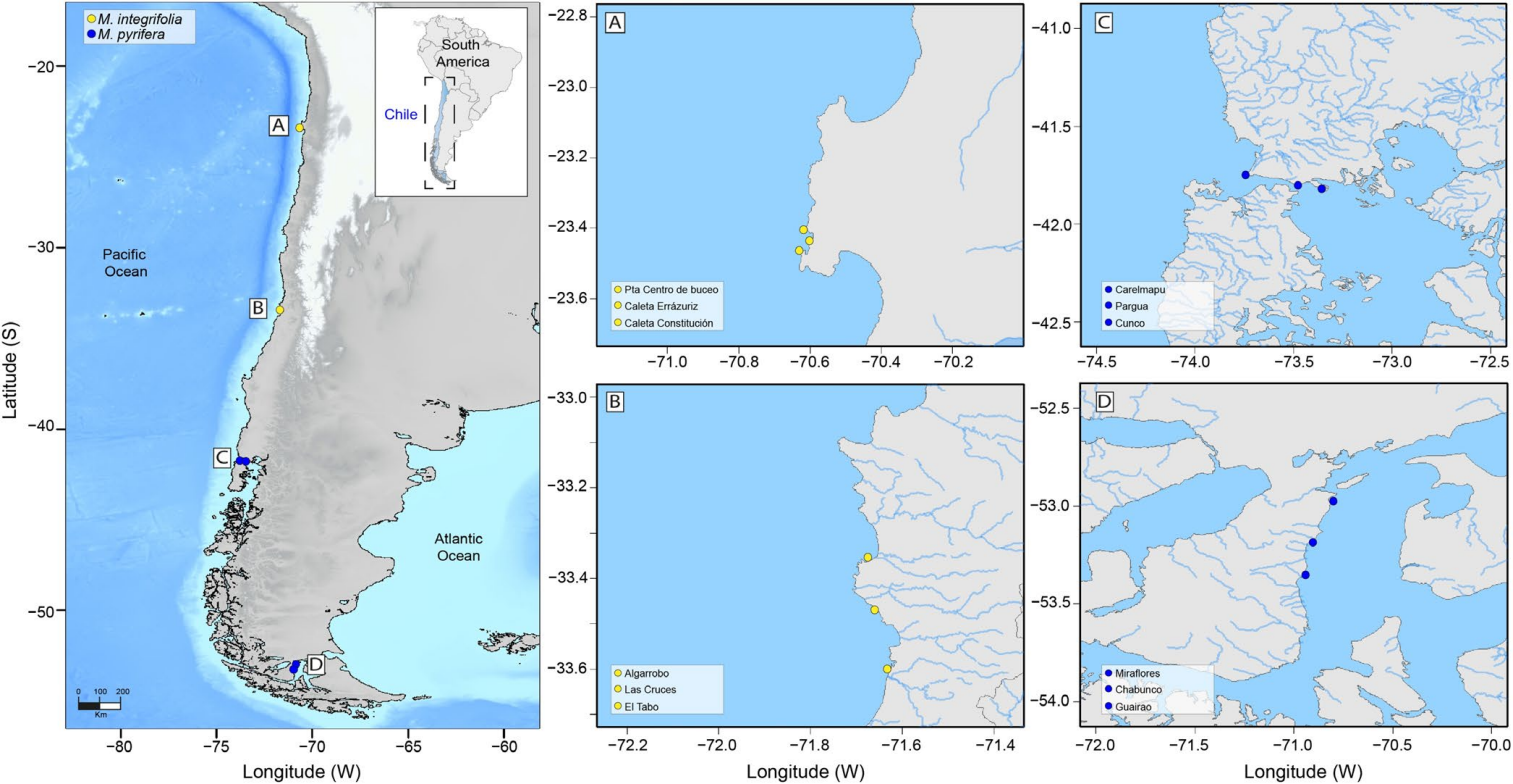
Map indicating the locations of the four study regions in the Chilean Coast showing the positions of the 3 study sites (red dots) within each region named from north to south covering a latitudinal gradient of ~3400 km. (A) Antofagasta (Punta centro de Buceo, Caleta Errázuriz, and Caleta Constitución). (B) Valparaíso (Algarrobo, Las Cruces, and El Tabo). (C) Los Lagos (Pargua, Carelmapu, and Cunco). (D) Magallanes (Miraflores, Chabunco, and Guairao).

**FIGURE 2:**
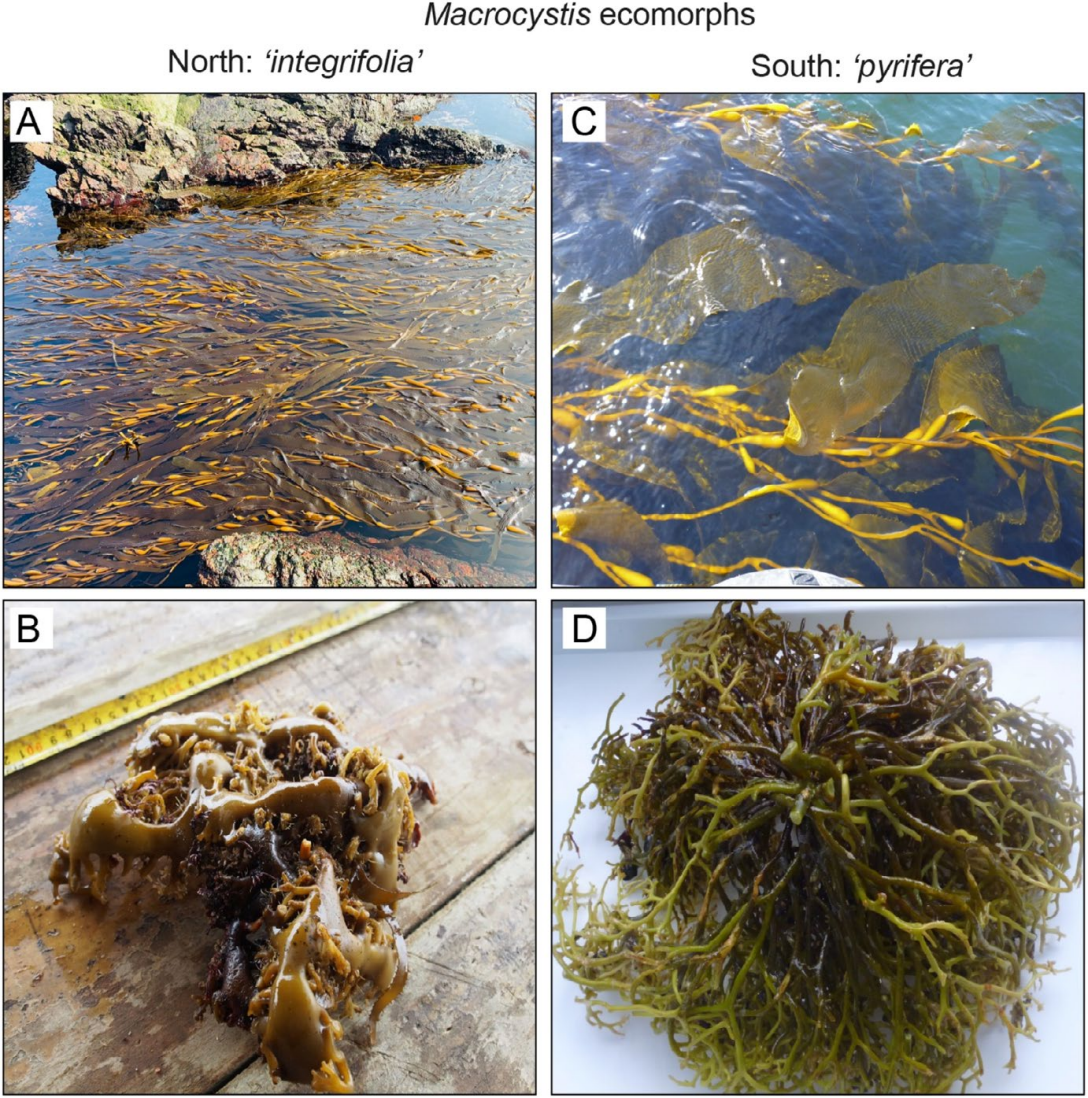
Morphological differences between both *Macrocystis* ecomorphs distributed in the Chilean coasts. Northern populations ( *M. integrifolia* ) blades/fronds (A) and holdfast (B). Southern populations ( *M. pyrifera* ) blades/fronds (C) and holdfast (D).

To compare the zoospore NL content and their functional responses of Chilean *Macrocystis*, fertile sporophylls of both ecomorphs “*integrifolia”* and *“pyrifera”* were collected at four different latitudes, from north to south (see Figure 1). Both ecomorphs were distinguished by the holdfast morphology, presenting “*integrifolia*” a rhizomatous and “*pyrifera*” a conical holdfast (Figure 2). Sporopylls from at least 15 independent sporophytes were collected at three sites separated at least by 30 km in 3 localities: Antofagasta, Valparaiso, Los Lagos, and Magallanes, which were separated at least by 30 km. The sporophylls were transported in ice, and after no more than 24 h, sporulation was induced in the laboratory using standardized and previously described methods (Gutiérrez et al. 2006), reaching a final concentration of 40,000 zoospores mL^−1^.

### 2.2 Samples for NL Determination Using a Flow Cytometer

Samples for flow cytometry analysis were prepared using collected zoospores of both ecomorphs from the 12 sampling sites (3 sites for each region, Figure 1). Each suspension was filtered using an 80 μm nylon mesh to remove coarser particles and collected in duplicate in 50 mL Falcon tubes (Thermo Scientific Nunc). Excessive polysaccharides from the sporophylls were removed by centrifugation (3200 rpm × 10 min), and zoospores were harvested and resuspended in 50 mL of filtered seawater (0.22 μm). This suspension was fixed at 1% formalin (Sigma‐Aldrich 37%) and 1% glutaraldehyde (Sigma‐Aldrich 25%) for at least 24 h and stored at 4°C until analysis.

To analyze *Macrocystis* zoospores NL, the fluorescent stain Nile Red was used in the Apogee A40 flow cytometer with two lasers (blue 488 nm and red 633 nm). The yellow/green fluorescence of Nile Red [9‐diethylamina‐5H‐benzo[a]phenoxazine‐5‐one] is specific for NL when excited at 488 nm and measured in the FL2 cytogram at 575 ± 15 nm (Brzezinski, Reed, and Amsler 1993; Reed et al. 1999). Formalin/glutaraldehyde fixed zoospores were collected by centrifugation (3200 rpm × 10 min) and washed twice with Phosphate Buffer Solution (PBS 1×, pH 7.0) to remove fixatives. After that, the pellets were stained with 200 μL of Nile Red (50 μg mL^−1^ solution in acetone) in 1.5 mL Eppendorf tubes for 2 h in dark, in duplicate. Afterward, to remove the Nile Red excess, the zoospore suspension was washed once as described above and resuspended in 250 μL of PBS, also in dark conditions. The autofluorescence emission (i.e., chlorophyll‐*a*, hereafter “chla”) from the same sample was used as zoospore density quantification, which was recorded in the FL3 cytogram emissions (Di Caprio et al. 2018; Urrutia, Yañez‐Mansilla, and Jeison 2019). Each sample was analyzed for 4 min with a fixed flow rate of 4.51 μL min^−1^ using logarithmic amplification of each fluorescence signal (Nile Red and chla, respectively). Finally, fluorescent calibration beads (5000 evt μL^−1^) for blue and red lasers were used before running each sample. The flow cytometer data from the Nile Red stained samples were finally expressed as relative fluorescence units in percentage (i.e., the ratio between the chla and Nile Red signals).

### 2.3 Confocal Microscopy for *M. pyrifera* NL Observations

To confirm the presence of *Macrocystis* NL, a zoospore fixed sample control from Los Lagos was stained for NL observations with 200 μL of Bodipy of 2 μM stock solution [4,4‐difluoro‐1,3,5,7,8‐pentamethyl‐4‐bora‐3a,4a‐diaza‐s‐indacene] in PBS 1× (excitation/emission wavelength 488/530 nm), for 30 min in the dark, following the procedures conducted for NileRed staining. Before the observation, the stained zoospores were harvested by centrifugation at 3200 rpm for 10 min to remove excess staining and resuspended in 20 μL of PBS 1×, and then mounted on slides and visualized in the Fluoview FV1000 Confocal Laser Scanning Microscope (Olympus, Japan).

### 2.4 Zoospore Germination

Zoospore germination success was determined by quantifying the density of newly formed germlings (germlings mm^−2^) after 48 h. Zoospore suspensions from all populations were kept under standardized culture conditions of light (20 ± 1 μmol photons m^−2^ s^−1^), photoperiod (16:8, L:D), and temperature (12°C ± 1°C), with complete Provasoli culture medium (McLachlan 1973) in 50 mL culture flasks (10,000 cells mL^−1^) to allow settlement and germination. Then, their density was registered and statistically compared.

### 2.5 Literature Review

A literature review of reproductive and developmental responses (by trait) of two *Macrocystis* ecomorphs ( *M. integrifolia* and *M. pyrifera* ) was conducted focusing on (i) Genetic differentiation; (ii) Zoospore production; (iii) Zoospore germination; (iv) Sexual reproductive success; (v) Juvenile sporophyte growth; (vi) Juvenile sporophyte performance; and, (vii) Juvenile sporophyte response to nutrient limitation. This information will be used to summarize the *Macrocystis* ecomorphs' early development and support their evolutionary trend.

### 2.6 Data Analysis

All data (NL and germlings) were analyzed using an ANOVA model I (Generalized Linear Model, GLM) with a log link function and negative binomial distribution (McCullagh and Nelder 1989) to evaluate the influence of population origin on zoospore NL and their germination. Previously, residual normality was tested graphically through qqplots, and residual homoscedasticity was tested with Levene's test (Fox and Weisberg 2011; Venables and Ripley 2010). Finally, Tukey's multiple comparison tests were run to determine differences between zoospore NL and germination in each region (Hothorn, Bretz, and Westfall 2008). The performed linear models were conducted using “car,” “lme4,” and “MASS” packages, available through the CRAN repository (http://www.r‐project.org). For all statistical tests, the significance level was set to *α* = 0.05, and each analysis was made using the statistical and programming software R 4.3.1 (R Core Team 2023). Median values and inter‐quartile range (IQR) were used to report the zoospore NL (%) and density of germlings (number mm^−2^).

## 3 Results and Discussion

**FIGURE 3:**
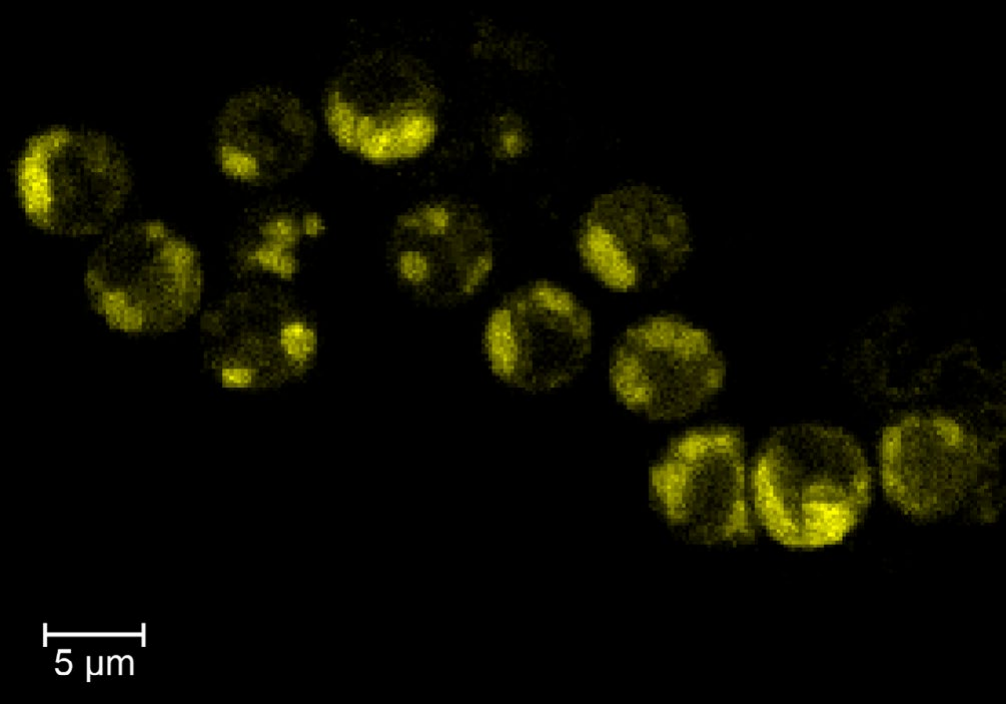
Zoospores stained with Bodipy for neutral lipids observed under confocal microscopy (see methods for further details).

The zoospores stained for confocal microscopy showed that the NL can be observed in fixed samples and, as described by Steinhoff et al. (2011) the NL represents nearly 1/3 of the zoospore size ~5 to 7 μM (Figure 3). Furthermore, the fixed/stained protocol developed maintains the zoospore integrity, avoiding their lysis, so these fixed cells can be used for NL flow cytometry analysis (Figure 3). This methodology differs from the Nile Red staining used by Reed et al. (1999) and the Sybr green is used for DNA staining by Müller et al. (2016). As *Macrocystis* NL can be detected in fixed zoospores, when testing in vivo is logistically impossible, as in our case where samples covered a latitudinal gradient (~3400 km). Thus, this methodology can be a valuable tool for large zoospore kelp samples. However, the effect of time storage must be determined, and care must be taken as preserving samples for longer times might underestimate NL in the zoospores.

**FIGURE 4:**
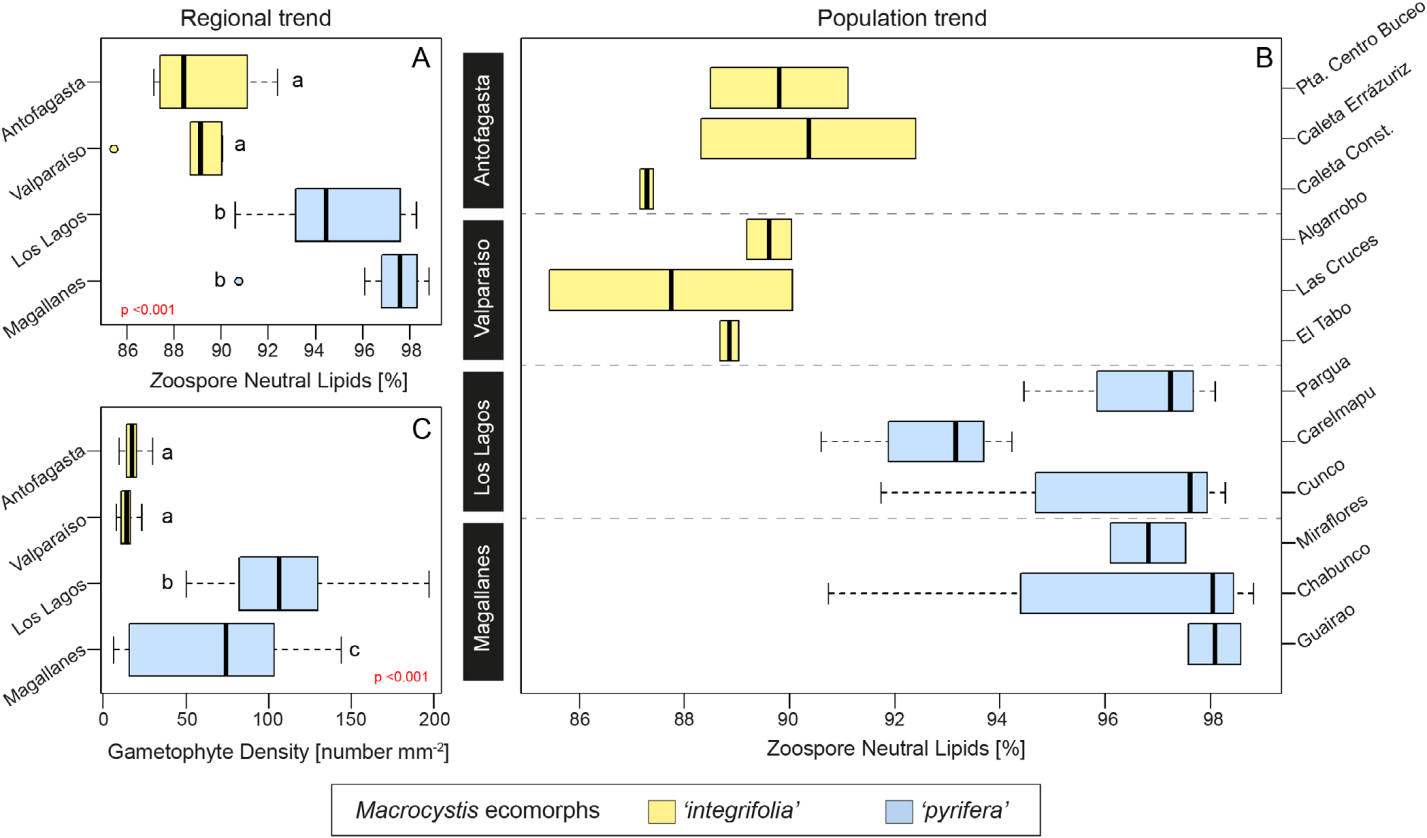
Regional and site‐specific trends of *M. pyrifera* zoospore neutral lipids and gametophyte density using latitudinal criteria, from the north (top) to the south (bottom). (A) Regional trend of zoospore neutral lipids. (B) Population trend of zoospore neutral lipids. (C) Regional trend of gametophytes density (mm^2^). The lowercase letters showed the results of Tukey's multiple comparison test. The boxplots show a range (whiskers), median (bold line), and interquartile range (box height).

The NL content in *Macrocystis* zoospores increased with latitude in a clear poleward trend (Figure 4A). The lowest NL values were found in “*integrifolia*” from Antofagasta with 88.40% (IQR = 2.81) and Valparaíso with 89.10% (IQR = 1.05), followed by “*pyrifera*” at Los Lagos and Magallanes with 94.44 (IQR = 4.44) and 97.57% (IQR = 1.50), respectively (Figure 4A). The NL content differed significantly among regions (*p* < 0.001) (Figure 4A; Table 1) showing that Antofagasta and Valparaiso are different (*p* < 0.05) compared to Los Lagos and Magallanes. Moreover, differences can be observed within each region, showing large intraspecific variability consistent with the phenotypic plasticity described for the species (Demes, Graham, and Suskiewicz 2009) as summarized in Figure 4B. For example, following the same poleward trend but under a population scope, the lowest median values of NL in zoospores were observed in Caleta Constitución (Antofagasta) with 87.27% (IQR = 0.13), followed by Las Cruces (Valparaíso) with 87.73% (IQR = 2.31), Carelmapu (Los Lagos) with 93.15% (IQR = 1.81), and Miraflores (Magallanes) with 96.81% (IQR = 0.71) (Figure 4B), but the highest median values were found in Caleta Errázuriz (Antofagasta) with 90.35% (IQR = 2.04), Algarrobo (Valparaíso) with 89.60% (IQR = 0.42), Cunco (Los Lagos) with 98.28% (IQR = 3.27), and Chabunco (Magallanes) with 98.04% (IQR = 4.04) (Figure 4B).

**TABLE 1:** Statistical significance of explanatory variables was determined using a *X* ^2^ test of a sequential (type I) ANOVA of the variability of the zoospore neutral lipids (A) and gametophytes density (B) in each region, using a generalized linear model (GLM) with a log link function for the residual negative binomial distribution.

	Predictive variables	df	Deviance	Residual df	Residual deviance	*Pr >* (|*X* ^2^|)
(A)	Null (Neutral Lipids)			27	485.70	
	Zone	3	334.83	24	150.88	< 0.001
(B)	Null (Gametophytes)			159	587.15	
	Zone	3	427.95	156	159.20	< 0.001

The success of zoospore germination also showed significant differences (*p* < 0.001) among kelp populations (Figure 4C). The Tukey test indicated similar densities for Antofagasta and Valparaiso, but differing significantly from Los Lagos and Magallanes, and also significant differences among Los Lagos and Magallanes were found (Table 1; Figure 4C). The density of germlings from the north populations was equally less dense for Antofagasta and Valparaíso with median values of 17.18 (IQR = 6.25) and 14.06 (IQR = 5.07) germlings mm^−2^, respectively (Figure 4C). Moreover, Los Lagos exhibited the highest density of germlings with a median of 106.25 (IQR = 44.14) followed by Magallanes with a median of 74.21 (IQR = 85.93) germlings mm^−2^ (Figure 4C).

The differences found among both ecomorphs in the allocation of NL in the zoospores and their success in developing into gametophytes and juvenile sporophytes added valuable information about the reproductive traits of northern and southern populations of *Macrocystis*. Hence, our results are consistent with the pattern of the genetic structure defined by Camus, Faugeron, and Buschmann (2018) and Gonzalez, Alberto, and Molano (2023), and the morphological variability previously described for this genus, indicating a reduced propagation of capacity of the “*integrifolia*” in comparison to the “*pyrifera*” ecomorph.

NL content in the zoospores and its germination capacity seems to have consequences on gametophytes and sexual reproduction traits according to Buschmann et al. (2004, 2006) and Camus et al. (2021), zoospore production, germination, gametophyte growth, and reproductive success are lower in “*integrifolia*” than “*pyrifera*” (Table 2). Furthermore, the “*pyrifera*” juvenile sporophytes also showed consistently better performance than “*integrifolia*” (Buschmann et al. 2004); however, juvenile sporophytes present on the Chilean coast of “*integrifolia*” seem more capable of dealing with limiting nitrogen events (Florez et al. 2021). Hence, the microscopic early developmental stages of juvenile sporophytes of both ecomorphs seem to be adapted to their specific local conditions (Solas et al. 2023). Nevertheless, a previous study did not find evidence of local adaptation of microscopic stages (Becheler et al. 2022).

**TABLE 2:** Reproductive and developmental responses (by trait) of two *Macrocystis* ecomorphs: *M. integrifolia* and *M. pyrifera* .

Population traits	*Macrocystis* ecomorphs		Comments/specific data	References
	*M. integrifolia*	*M. pyrifera*		
Genetic differentiation	Yes	Yes	Populations are genetically structured	Camus, Faugeron, and Buschmann (2018), Gonzalez, Alberto, and Molano (2023)
Zoospore production	Lower	Higher	*M. integrifolia* zoospore production remains lower across the year, while the *M. pyrifera* population is reproductive all year round reaching higher zoospore production values in summer and fall	Buschmann et al. (2004, 2006)
Zoospore germination	Lower	Higher	*M. integrifolia* zoospore germination reaches ≤ 25%, while *M. pyrifera* ranges to 75%–100%, of the total available zoospore	Buschmann et al. (2004)
Sexual reproductive success	Lower	Higher	The number of oogonia per female gametophyte is three times higher in *M. pyrifera* (max. 3.5 oogonia female gametophyte^−1^) than in *M. integrifolia* (max. 1.0 oogonia female gametophyte^−1^)	Camus et al. (2021)
Juvenile sporophyte growth	Lower	Higher	*M. integrifolia* 0.02 mm month^−1^; *M. pyrifera* 0.06 mm month^−1^	Buschmann et al. (2004)
Juvenile sporophyte performance	Lower	Higher	*M. pyrifera* showed a reduced reproductive success associated with outbreeding, while *M. integrifolia* favored hybrid crosses.	Solas et al. (2023)
Juvenile sporophyte response to nutrient limitation	Higher	Lower	Juvenile sporophytes of *M. pyrifera* has a lower specific growth rate (SGR) under N‐limiting in comparison with *M. integrifolia*	Florez et al. (2021)

Hence, all the evidence compiled and the molecular data shown (Gonzalez, Alberto, and Molano 2023) suggest that *Macrocystis* ecomorphs “*pyrifera*” and “*integrifolia*” might be following a speciation trend. Westermeier, Patiño, and Müller (2007) and Murúa et al. (2020) showed that both *Macrocystis* ecomorphs can hybridize under laboratory conditions. Still, as intrafamily kelp genera can hybridize (Lewis and Neushul 1994) and interfamily hybridization can occur in nature (Murúa et al. 2020), this evidence is inconclusive. Interestingly, unpublished results by the authors show restricted hybridization between the northern and southern populations, resulting in an F1 hybrid generation of sporophytes with a predominant genotype belonging to the southern populations. This might indicate that inter‐ecomorph breeding is not entirely successful. Furthermore, it has been shown that the F1 hybrid ecomorph sporophytes perform better at higher temperatures (Murúa et al. 2020), suggesting that the genetic exchange between populations produces a genetic variation. In addition to all this evidence, Gonzalez and Raimondi (2024) demonstrated that the morphological features that distinguish both morphotypes are genetically determined rather than environmentally regulated, as previously claimed by Demes, Graham, and Suskiewicz (2009). By using cultivated sporophytes obtained from both morphotypes in California and outplanted to a common garden experimental setup. Similar studies to separate genetic from environmental factors must be undertaken together with demonstrating that a 2nd generation could inherit NL production capacity. Overall, our results, along with previous studies, indicate that both genetically distinct ecomorphs show differential morphological and reproductive functional traits, supporting the hypothesis of separating two divergent lineages in the southern hemisphere.

## Author Contributions

**Camilo Rodríguez‐Villegas:** conceptualization (equal), data curation (equal), formal analysis (equal), investigation (equal), methodology (equal), software (equal), visualization (equal), writing – original draft (equal), writing – review and editing (equal). **Alejandro H. Buschmann:** conceptualization (equal), data curation (equal), formal analysis (equal), funding acquisition (equal), investigation (equal), methodology (equal), project administration (equal), software (equal), supervision (equal), validation (equal), visualization (equal), writing – original draft (equal), writing – review and editing (equal). **Mayra A. Barrios:** data curation (equal), formal analysis (equal), investigation (equal), methodology (equal), writing – original draft (equal). **Sandra Pereda:** conceptualization (equal), data curation (equal), formal analysis (equal), investigation (equal), methodology (equal), resources (equal), supervision (equal), validation (equal), visualization (equal), writing – original draft (equal), writing – review and editing (equal). **Carolina Camus:** conceptualization (equal), data curation (equal), formal analysis (equal), funding acquisition (equal), investigation (equal), methodology (equal), project administration (equal), supervision (equal), validation (equal), visualization (equal), writing – original draft (equal), writing – review and editing (equal). **Pamela Fernández:** formal analysis (equal), investigation (equal), methodology (equal), validation (equal), visualization (equal), writing – original draft (equal), writing – review and editing (equal). **María C. Hérnandez‐González:** conceptualization (equal), funding acquisition (equal), investigation (equal), project administration (equal), resources (equal), supervision (equal), validation (equal), writing – original draft (equal). **Ángela M. Baldrich:** data curation (equal), formal analysis (equal), investigation (equal), software (equal), visualization (equal), writing – review and editing (equal). **Cynthia Urrutia:** data curation (equal), formal analysis (equal), methodology (equal), validation (equal), writing – review and editing (equal). **Ailen M. Poza:** formal analysis (equal), investigation (equal), methodology (equal), validation (equal), visualization (equal), writing – original draft (equal), writing – review and editing (equal). **Karina Villegas:** formal analysis (equal), investigation (equal), methodology (equal), resources (equal), software (equal), supervision (equal), validation (equal), visualization (equal), writing – original draft (equal), writing – review and editing (equal). **Camila Martínez:** data curation (equal), formal analysis (equal), investigation (equal), methodology (equal), visualization (equal), writing – review and editing (equal). **Jaime Vargas:** data curation (equal), formal analysis (equal), investigation (equal), methodology (equal), resources (equal), software (equal), writing – original draft (equal), writing – review and editing (equal).

## Conflicts of Interest

The authors declare no conflicts of interest.

## Data Availability

Raw data: figshare, public with https://figshare.com/s/b46bf37f6df275229472.
